# MicroRNA expression profiles in peri-miniscrew implant crevicular fluid in orthodontics: a pilot study

**DOI:** 10.1186/s12903-021-02009-w

**Published:** 2021-12-18

**Authors:** Wendan He, Yanru Yang, Longgan Cai, Qiaoling Lei, Zhongdong Wang, Xiaoxia Che

**Affiliations:** 1 Capital Medical University School of Stomatology, Beijing, 100006 China; 2 Department of Stomatology, Shenzhen Hospital, Southern Medical University, Studenthen, Guangzhou, 518033 China; 3 Chi-Biotech Co. Ltd., Shenzhen, 518023 China; 4 Beijing Stomatological Hospital, Capital Medical University, Beijing, 100006 China; 5 Capital Medical University School of Stomatology, Temple of Heaven Xili 4, Dongcheng District, Beijing, 100000 China

**Keywords:** MicroRNA/miRNA, PMICF, GCF, Peri-implantitis, Biomarker, Orthodontics

## Abstract

**Background:**

This study systematically evaluated microRNA (miRNA) expression patterns in peri-miniscrew implant crevicular fluid (PMICF) in orthodontic patients.

**Methods:**

Next-generation sequencing (NGS) was performed to obtain miRNA profiles in PMICF or gingival crevicular fluid (GCF) collected from 3 healthy volunteers (H), 3 peri-implantitis patients (PMSII) and 5 periodontitis patients (P). MiRNA expression patterns were compared between normal and orthodontic PMICF and GCF. Differentially expressed miRNAs were estimated by quantitative real-time PCR (qRT-PCR). Enrichment analyses of the gene targets controlled by these miRNAs were conducted by Gene Ontology (GO) enrichment and Kyoto Encyclopedia of Genes and Genomes (KEGG) pathway analyses.

**Results:**

Compared with healthy donors, in PMSII patients, a total of 206 upregulated miRNAs and 152 downregulated miRNAs were detected in PMICF, while periodontitis patients had 333 upregulated miRNAs and 318 downregulated miRNAs. MiR-544a, miR-1245b-3p, miR-1825, miR-4291, miR-3689e, and miR-4477a were chosen randomly for further examination. qRT-PCR examination confirmed that the expression levels of miR-1245b-3p and miR-4291 were higher in PMSII than in H samples and that the expression levels of miR-1825 were higher in PMSII than in P samples. However, contrary to the NGS results, qRT-PCR analysis showed decreased expression of miR544a in PMSII. MiR3689e and miR4477a expression did not differ significantly among all samples. According to GO and KEGG pathway analyses of miR-1825, miR-4291, and miR-1245b-3p high enrichment of target genes involved in the PI3K-AKT signalling pathway was observed.

**Conclusions:**

The NGS analysis of normal and orthodontic PMICF/CGF showed different miRNA profiles, which may lay the foundation for future research on the molecular mechanism of PMSII. miR-4291, miR-1245b-3p and miR-1825 may be used as diagnostic markers and potential therapeutic targets for PMSII.

## Background

Malocclusion is a common disease in stomatology and has been identified by the World Health Organization (WHO) as one of the three major oral diseases. The prevalence of this disease has been estimated to include almost 60–70% of the population in China. In recent years, the use of implant therapy has been widely accepted and chosen as a routine procedure for the reconstruction of fully or partially edentulous individuals due to the advantages of small size, a simple operation, a small surgical wound, a flexible implant site, and high tolerance. According to recent reports, the survival rates of miniscrew implants used for orthodontic anchorage range from 57 to 95.3%, with an average of approximately 84% [1, 2]. The increasing need for implants leads to a significant rise in the number of individuals with posttreatment complications.

Peri-implantitis is an inflammatory response surrounding implants that will cause the loss of the supporting bone in the tissues surrounding a functioning implant and even loss of the anchorage. It has been shown that peri-implantitis can affect long-term success following the osseointegration process and can affect the stability of established implants [3, 4]. The mean implant-based and subject-based peri-implantitis prevalence was 9.25% and 19.83%, respectively [5]. The diagnosis of peri-implantitis is generally through clinical symptoms and imaging examinations. Peri-implant crevicular fluid (PMCF), derived from the exudate of plasma and tissue fluid, can penetrate into the implant anchorage and attached gingiva through periodontal connective tissue and has a similar composition to gingival crevicular fluid (GCF). It contains a variety of inflammatory mediators and bioactive substances and is considered a convenient medium to study PMSII [6–9].

MicroRNAs (miRNAs) are small noncoding RNAs that regulate gene expression by binding to complementary sequences in the 3′-untranslated or coding regions of target mRNAs, leading to gene silencing [10, 11]. They are critical regulators of the host immune and inflammatory response against bacterial pathogens [12, 13]. Previous reports have shown that a large number of microRNAs can be detected in GCF and that miRNAs exert control over all aspects of innate and adaptive immunity in periodontal disease, which suggests the potential role of miRNAs as biomarkers and therapeutics for PMSII [14–16]. However, most available miRNA expression information about PMSII was obtained from experimental animals [16] due to difficulty in sampling and small sample size. Therefore, Analysis the PMSII miRNA expression information in clinical samples is of great significance for miRNAs as PMSII biomarkers and potential therapeutic targets.

In this study, we used miRNA sequencing to identify differentially expressed miRNAs in peri-implantitis patients and validated the results using real-time PCR. The target genes regulated by differ-miRNAs were located by bioinformatic tools, and the corresponding signaling pathway identification and cluster analysis were performed. Based on the results of the analysis, some miRNAs were selected as candidate molecular targets related to peri-implantitis. In conclusion, this study aims to clarify the miRNA expression profile of PMSII, explore the molecular mechanism of PMSII, and lay the foundation for the targeted therapy and clinical diagnosis of PMSII.

## Methods

### Clinical subjects

Three healthy volunteers (H), three PMSII patients (PMSII), and five periodontitis patients (P) were recruited for sampling of PMCF/GCF used for comprehensive miRNA profiling. The samples were collected from oral patients admitted to Shenzhen Hospital of Southern Medical University from January 2019 to June 2019. There was no significant difference in the general information among the three groups (all *p* > 0.05). The inclusion and exclusion criteria of the PMSII patients: Patients with peri implant anchorage inflammation caused by implant anchorage were diagnosed according to clinical symptoms; No periodontal treatment or other related systematic treatment was performed within 1 year; There was no history of taking antibiotics and other oral diseases within 3 months. No smoking; Women are not in pregnancy; All patients were treated in permanent dentition with straight wire arch technique; Patients with systemic diseases, infectious diseases, allergic constitution and poor compliance were excluded. The inclusion and exclusion criteria of the periodontitis patients: Patients diagnosed with periodontitis according to the diagnostic criteria of periodontitis [17]; No periodontal treatment or other related systematic treatment was performed within 1 year; There was no history of taking antibiotics and other oral diseases within 3 months; No smoking; Women are not in pregnancy; All patients were treated in permanent dentition with straight wire arch technique; Patients with systemic diseases, infectious diseases, allergic constitution and poor compliance were excluded. Inclusion criteria for healthy volunteers: After oral examination, there were no oral diseases such as periodontitis and gingivitis; Did not receive orthodontic treatment and implant treatment within 1 year; No systemic disease or infectious disease; Women are not in pregnancy; All volunteers received oral hygiene education from professional medical staff and used the same toothpaste to brush their teeth three times a day; There was no history of taking antibiotics and other oral diseases within 3 months. No smoking. The basic information of samples is shown in Table 1. This study was approved by the Research Ethics Committee in Shenzhen Hospital of Southern Medical University. All ethical procedures conformed to the principles of 1964 Declaration of Helsinki and its latest 2008 amendments. All persons gave their informed consent prior to their inclusion in the study.

**Table.1:** The basic information of samples

**Group**	**Sample ID**	**Gender**	**Age**	**Clinical presentation**	**Sampling location**	**Filter strip** (mm * mm)
H	H1	Female	44	No abnormal symptoms	15/16	15 × 5
H	H2	Female	27	No abnormal symptoms	15/16	15 × 5
H	H3	Female	37	No abnormal symptoms	15/16	15 × 5
P	P1	Female	24	Red and swollen gums, periodontal pocket depth = 4.5 mm, Stage II	14/15	15 × 5
P	P2	Female	27	Gingival bleeding, periodontal pocket depth = 4.6 mm, Stage II	15/16	15 × 5
P	P3	Female	23	Red and swollen gums, periodontal pocket depth = 4.3 mm, Stage II	15/16	15 × 5
P	P4	Male	15	Red and swollen gums, periodontal pocket depth = 4.2 mm, Stage II	15/16	15 × 5
P	P5	Female	25	Red and swollen gums, periodontal pocket depth = 4.5 mm, Stage II	13/14	15 × 5
PMSII	PMSII-1	Female	27	implant anchorage the surrounding soft tissue swelling and easy bleeding	24	15 × 5
PMSII	PMSII-2	Male	15	implant anchorage the surrounding soft tissue redness and swelling	25	15 × 5
PMSII	PMSII-3	Female	25	Implant anchorage loosening, and the surrounding soft tissue redness and swelling	34	15 × 5

### Peri-implant crevicular fluid (PMCF) or gingival crevicular fluid (GCF) collection

Sampling process was performed as previously described [17]. Prior to sampling, the supragingival plaque and saliva around teeth or implants were removed using a cotton pellet, and the teeth or implants were air-dried and isolated with cotton rolls. GCF samples was collected with Microcapillary tubes (Sigma-Aldrich, USA), which were gently inserted into the entrance of the sulcus/periodontal pocket and left in place for no more than 10 min. Multiple tooth sites were used for collecting, in order to obtain sufficient samples. Any tube or sample visibly contaminated with blood was discarded. The samples of each group were pooled in sterilized tubes, snap frozen, and subsequently used for RNA isolation.

### RNA isolation

Total RNA was extracted from GCF using Trizol reagent (Invitrogen, Carlsbad, CA, USA). Quality of the RNA were determined by measuring the A260/A280 ratios using Bioanalyzer 2100 (Agilent, CA, USA). RNA samples were immediately frozen and stored at −80 °C prior to use.

### miRNA sequencing

We generated miRNA libraries for deep sequencing as QIAseq^TM^ miRNA Library Kit (QIAGEN, Germantown, MD, USA) protocol described using 200ng of total RNA. This process included following steps: (1) 3′ adaptor ligation; (2) 5′ adaptor ligation; (3) cDNA synthesis; (4) PCR amplification; (5) libraries qualification using an Agilent 2100 Bioanalyzer (Agilent Technologies Inc., Wilmington, DE, U.S.A.). The libraries were sequenced by Illumina Hiseq2500 SE50 following the vendor’s recommended protocol. Data processing followed the established procedures. Briefly, the raw reads were subjected to Barcode (demultiplex). The dataset was further processed with cutadapt (version 1.15) to remove adapter dimers, junk, low complexity, common RNA families (rRNA, tRNA, snRNA, snoRNA), and repeats. Subsequently, unique sequences>17 nt in length were mapped to mature species in miRbase (Release 22.1) by FANSe3 to identify known miRNAs and novel 3p- and 5p- derived miRNAs. Length variation at both 3′ and 5′ ends and one mismatch inside of the sequence were allowed in the alignment.

### Differential expression analysis

miRNA differential expression, based on normalized deep-sequencing counts, was analyzed by selectively using edger (edgeR package in R) according to the experimental design. The fold change at log2 scale was set to be > 1 or < − 1 and significance threshold (*p* value) was 0.05.

### Quantitative real-time PCR (qRT-PCR) analysis

qRT-PCR was performed as previously described. Briefly, the purified 100ng total RNA was reverse transcribed into cDNAs using the TaqMan™ MicroRNA Reverse Transcription Kit (Invitrogen, Carlsbad, CA, USA) and qRT-PCR was performed using qRT-PCR SYBR Green Kit (Vazyme Biotech, NJ, China) according to the manufacturer’s instructions. The relative gene expression levels of miRNAs were evaluated using U6 as the endogenous normalization control. The primer of U6, forward, 5′- CTCGCTTCGGCAGCACA-3′, reverse, 5′-AACGCTTCACGAATTTGCGT-3′. The primer sequences used for the evaluated genes are listed in Table 2. All tests were performed in triplicates.

**Table.2:** qRT-PCR primers of chosen miRNAs

Primers	Sequences
hsa-miR-4477a	ATTAAGGACATTTGTGATTCCTCAA
hsa-miR-3689e	ATATCATGGTTCCTGGGACTCA
hsa-miR-544a	TCTGCATTTTTAGCAAGTTCCTC
hsa-miR-4291	AGCAGGAACAGCTCTCAACTGA
hsa-miR-1825	CCTCCTCTCCCTCAACTGAATT
hsa-miR-1245b-3p	GTTGTCAGATGATCTAAAGGCCTAT

### Analysis for target genes of the selected miRNAs

To predict the genes targeted by miRNAs, miRTarBase 7.0 was used to identify miRNA binding sites. Potential target genes were then analyzed by clusterProfiler program (Yu et al. 2012). Gene Ontology (GO) term enrichment and Kyoto Encyclopedia of Genes and Genomes (KEGG) pathway analysis were applied for the identification of key pathways regulated by selected miRNAs.

### Statistics

Cycle threshold (Ct) values were processed by LightCycler 480 v1.5.0.39 Software. The expression value of miRNAs relative to internal controls were calculated using the 2^−△△Ct^ method. Statistical analysis was performed with SPSS software. Data were tested for significance with the nonparametric Mann-Whitney U test. A P value < 0.05 was considered to be significant. A *p* value < 0.01 was considered to be extremely significant. Pearson correlation analysis was performed by SPSS 22.0.

## Results

### Different miRNA profiles in PMICF of PMSII and periodontitis patients

**Fig. 1:**
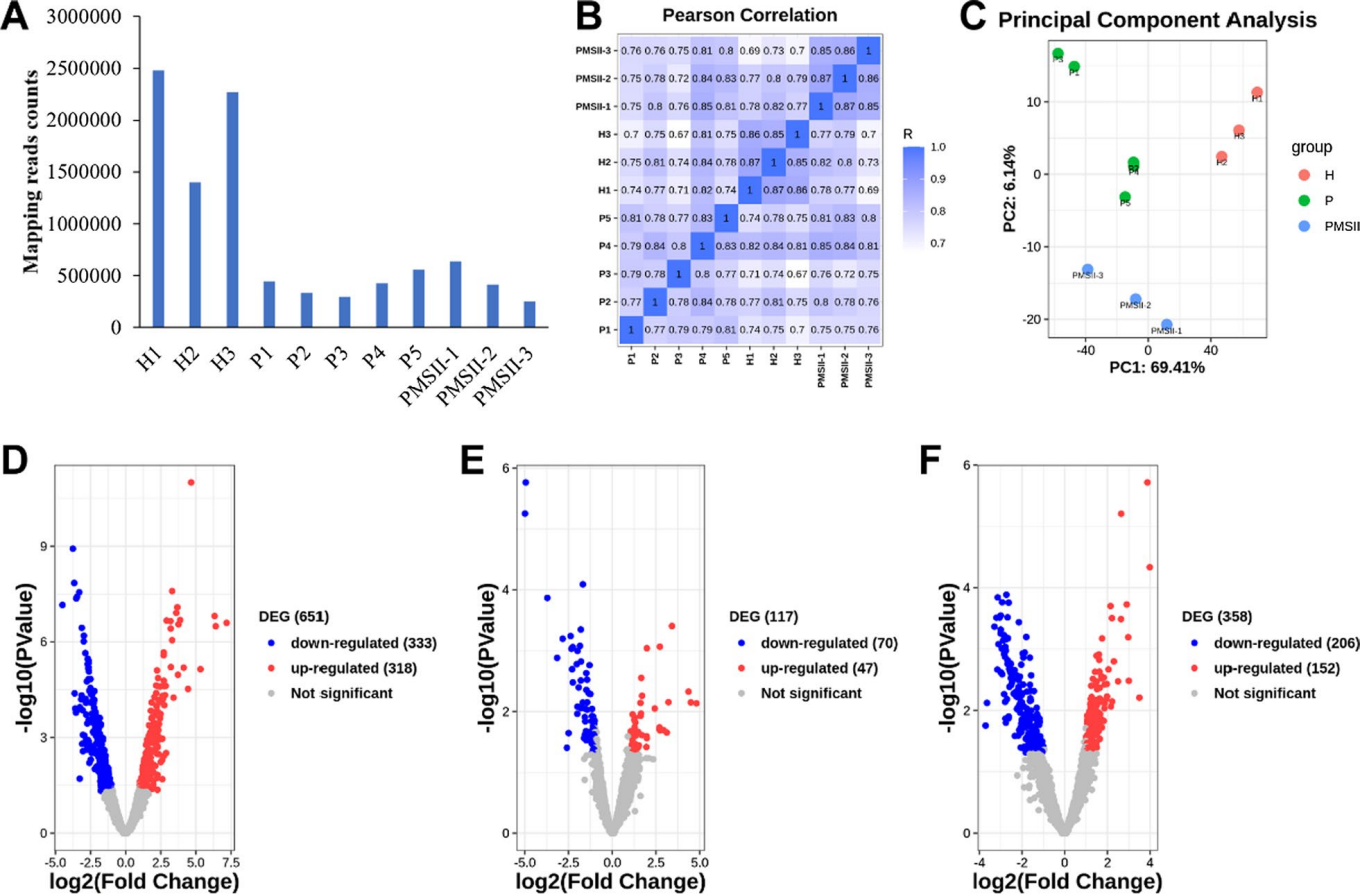
miRNA-sequencing of all samples. **A** The number of reads was mapped to human genome. **B** The Pearson correlation analysis between samples. **C** The principal component analysis of samples. Volcano maps of differentially expressed miRNAs in PMSII compared with H (**D**), PMSII compared with P(E), and P compared with H(F). H, samples of healthy volunteers. P, samples of periodontitis patients. PMSII, samples of patients with PMSII

Three PMSII patients (PMSII), five periodontitis patients (P), and three healthy volunteers (H) were analysed in this study. First, we successfully sequenced all sample miRNAs by using high-throughput sequencing. The number of reads mapped to the human genome ranged from 252,813 to 2,479,443 (Fig. 1a). According to the results of Pearson correlation analysis and principal component analysis (Fig. 1b, c), the correlation within the group is good, and can clearly distinguished between these three groups. Based on the criteria described before (|log2 (fold change)| >1 and *p* value < 0.05), 651 miRNAs were found to be differentially expressed in PMSII when compared to H (Fig. 1d). Among these miRNAs, 333 were upregulated and 318 were downregulated. Most of the differentially expressed miRNAs are associated with the inflammatory response and bone metabolism. In addition, 117 miRNAs were also found to be differentially expressed in PMSII compared to periodontitis (Fig. 1e), 358 miRNAs were also found to be differentially expressed in periodontitis compared to healthy samples (Fig. 1f).

### Identifying specific miRNAs associated with PMSII

**Fig. 2:**
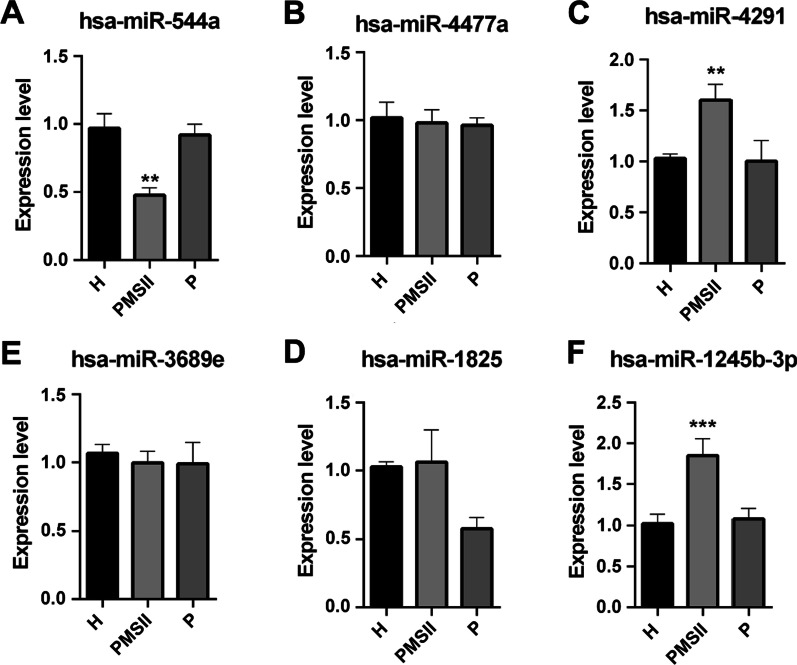
Verification of the several differentially expressed miRNAs by qRT-PCR. Relative Expression level of miR-544a**(A)**, miR-4477a**(B)**, miR-4291**(C)**, miR-3689e**(D)**, miR-1825**(E)** and miR-1245b-3p**(F)** in each group. **p < 0.01, ***p < 0.001 versus healthy group

To identify specific differentially expressed miRNAs in PMSII, we further compared miRNA expression patterns in PMSII with periodontitis. We found 476 miRNAs that were up- or downregulated in PMSII (|log2 (fold change)| > 1 and *p* value < 0.05) but had little change in periodontitis. To further verify the findings from analysing the miRNA profile, four miRNAs (miR544a, miR1245b-3p, miR1825, miR4291, miR3689e, and miR4477a) were chosen randomly to be verified by qRT-PCR (Fig. 2). Meanwhile, we chose two miRNAs as internal controls, which had the same expression pattern in both PMSII and periodontitis when compared to healthy samples. The results showed that the expression levels of miR-1245b-3p and miR-4291 were higher in PMSII than in H samples and that the expression levels of miR-1825 were higher in PMSII than in P samples. However, contrary to the NGS results, qRT-PCR showed decreased expression of miR544a in PMSII. MiR3689e and miR4477a expression was not significantly different among all samples (Fig. 2; Table 3).

**Table.3:** NGS results of selected miRNAs

miRNA	Fold-change (PMSII/H)	*p* Value	Fold-change (P/H)	*p* Value
miR544a	1.8941	0.04051	−0.0432	0.9958
miR1245b-3p	1.9698	0.0296	1.2743	0.1962
miR1825	1.6333	0.0101	None	None
miR3689e	1.0354	0.2903	1.3875	0.1260
miR4291	2.6120	0.0202	None	None
miR4477a	0.8111	0.2547	None	None

### Target prediction and functional annotations

**Fig. 3:**
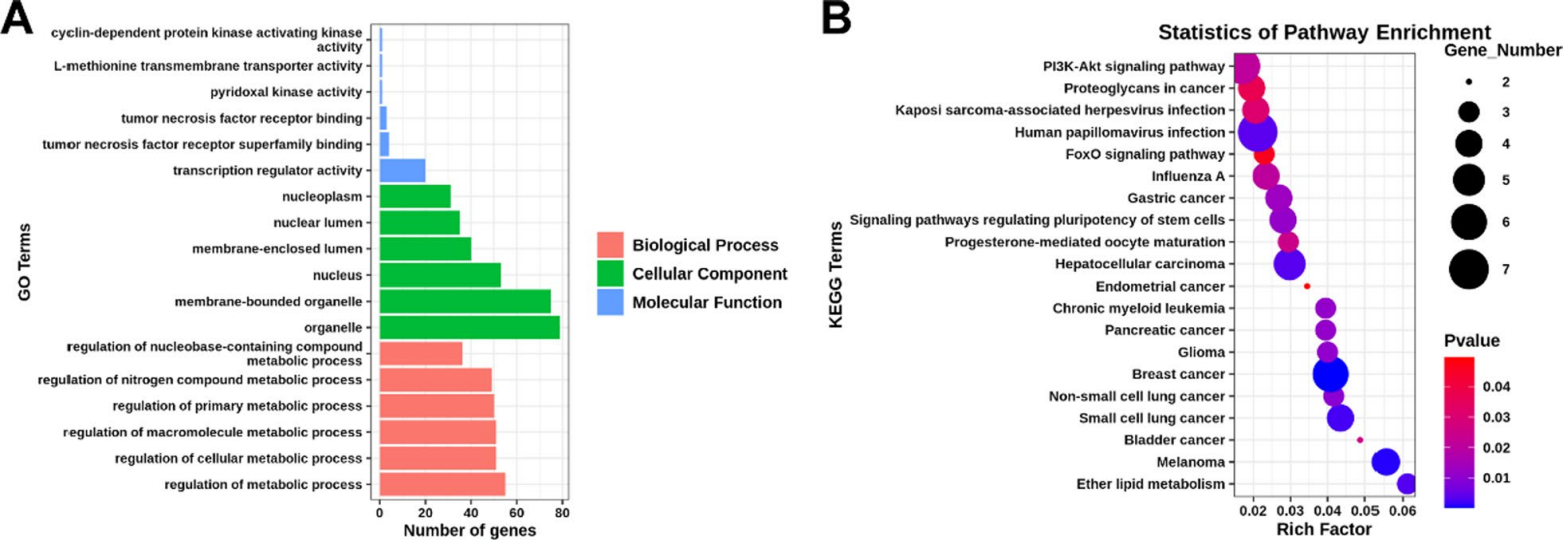
GO and KEGG pathway analyses of the target genes of miR4291, miR1825 and miR-1245b-3p. **A** Top 6 most significantly enriched terms of GO. **B** KEGG analysis of top 20 pathways

To understand the biological functions of miR4291, miR1825 and miR-1245b-3p, we next performed GO and KEGG pathway analyses using miRTarBase Release 7.0. We chose the top 6 most significantly enriched terms (*p* < 0.05, FDR ≤ 25%) to draw a pie chart and bar chart (Fig. 3a). As shown in Fig. 3a, in addition to the regulation of metabolic processes, the regulation of macromolecular metabolic processes, which are related to macrophage differentiation [19, 20], was associated with the greatest number of genes.

For KEGG pathway-enrichment analysis, we found 79 statistically significant (*p* < 0.05) terms (not shown). The 20 pathways that exhibited a smaller *p*-value are shown in Fig. 3b, The analysis indicated that the signaling pathways with significant correlations included PI3K-Akt signalling pathway and FoxO signalling pathway. These pathways are known to be closely related to inflammation regulation and osteoclast activity, suggesting that miRNAs in PMCFs may affect the inflammatory process through intricate molecular signals.

## Discussion

PMSII is a destructive inflammatory disease affecting the soft and hard tissues around dental implants. Currently, the diagnosis and classification of PMSII mainly rely on conventional clinical assessments. The main clinical features include bleeding, sometimes suppuration when the probing pocket depth is increased, and radiographic examination of bony defects around implants [2]. Although peri implant infections are well described histopathologically, the molecular mechanism of these infections has not been fully determined. MiRNA participates in a variety of physiological and pathological mechanisms and plays an important role in the development of inflammatory response and diseases, including periodontitis and PMSII. Previous reports found that miR-146a may be a genetic determinant of an increased risk of PMSII by using blood samples [18], which was consistent with our miRNA profiling results. A miRNA sequence analysis of canine tissue flaps identified 8 upregulated and 30 downregulated miRNAs in PMSII. Among them, according to the author, let-7 g, miR-27a, and miR-145 may play important roles in PMSII, and their target genes are involved in the MAPK signalling pathway and regulate macromolecular biosynthesis processes and nitrogen-containing compound metabolic processes[19].

To date, our study is the first to use GCF, an exudate of periodontal tissues, to verify differentially expressed miRNAs in PMSII. Sample collection can be performed easily and quickly with a minimally invasive procedure [14, 20]. Because of their small size and low abundance, the detection of miRNAs in body fluids has been technically hampered. However, small RNA sequencing has been developed, and it can be highly accurate and sensitive in quantitating gene expression. Hence, by employing miRNA-seq, we identified 274 upregulated and 202 downregulated miRNAs in the GCF of PMSII, which had little change in periodontitis. These miRNAs are likely to promote inflammation or attempt to downregulate immune activation and bone resorption in PMSII lesions. We further selected 6 miRNAs and measured their expression using qRT-PCR to confirm their differential expression pattern. MiR-1825, miR-4291 and MiR-1245b-3p were consistent with the data obtained from the miRNA sequence analysis, the expression levels of miR-1245b-3p and miR-4291 were higher in PMSII than in H samples and that the expression levels of miR-1825 were higher in PMSII than in P samples. It suggests that they may act as biomarkers for PMSII.

To understand the functional effect of miR-1825, miR-4291 and MiR-1245b-3p, we performed GO and KEGG pathway analyses to identify the enriched pathways and functions of the target genes. Based on GO analysis, two of the most enriched functions of biological process were the regulation of metabolic processes and regulation of macromolecular metabolic processes. Macromolecular metabolism plays an important role in the immune system. Host defence in vertebrates is mediated by different secreted proteins (such as antibodies, complement, and antimicrobial peptides) and diverse leukocytes with distinct functions [21]. The GO results in this study suggested the potential role of miR1825 and miR4291 in regulating immune-related gene expression. Several studies have considered the particularly important role of class I PI3K, in which we found high enrichment of target genes involved in the immune system [22]. There is now a substantial body of evidence suggesting that PI3K regulates chemokine-mediated recruitment and activation of immune cells in innate immunity and regulates B and T lymphocyte development and function in adaptive immunity [21, 23]. Class I PI3Ks are always activated alongside other intracellular signalling pathways. The forkhead box O (FOXO) family of transcription factors regulates the expression of genes involved in cellular physiological events, including apoptosis, cell cycle control, glucose metabolism, oxidative stress resistance and immunoregulation. By activating class I PI3Ks, Akt can phosphorylate Foxo1, Foxo3 and Foxo4, leading to their nuclear exclusion and degradation, and their tagged genes can be turned off, such as IL-7R and Rag recombinases, which are essential for B cell development and Treg differentiation [24, 25]. The FOXO signalling pathway also showed high enrichment of target genes in our KEGG analysis. This implied the molecular mechanism by which miRNAs regulate PMSII. The results suggest that miR-4291, miR-1245b-3p and miR1825 may have important value in PMSII, and further mechanistic studies will be needed to dissect the roles of miRNAs in PMSII homeostasis and pathology.

MiR-1245b-3p was upregulated in both peri-implantitis and periodontitis, which was confirmed by qRT-PCR. There are few studies regarding miR-1245b-3p. A study of gastric cancer suggests that miR-1245b-3p can directly target the 3′UTR of GKN1 [26]. Differential expression of miRNA in guinea pigs infected with HSV2v found that miR-1245b-5p was increased and the downstream TLR pathway-associated genes were downregulated [27]. A study on the drug resistance of imatinib in BCR-ABL chronic myeloid leukaemia observed significant changes in the expression of miR-1245b-3p [28]. This study would prompt the future directions for miR-1245b-3p studies in the chronic myeloid leukaemia pathway in PMSII.

Our study is the first in the literature to examine miR544a in peri-implant and periodontitis. However, unfortunately, the verification result of qRT-PCR was inconsistent with the sequencing result, which may be due to the long storage time of this sample. In existing research, Stadio et al. found that GKN1 expression in gastric cancer cells is negatively regulated by miR-544a [26]. LEF1-AS1 contributes to proliferation and invasion by regulating the miR-544a/FOXP1 axis in lung cancer [29]. MiR-544a stimulates endometrial carcinoma growth via targeted inhibition of reversion-inducing cysteine-rich protein with Kazal motifs [30]. MiRNA-544a regulates the inflammation in spinal cord injury by inhibiting the expression of NEUROD4 [31] These results provide a good reference for the further study of miR544a in peri-implantitis or periodontitis.

Although the specific role of miRNAs in the occurrence and development of peri-implantitis and periodontitis is still poorly understood, the research and application prospects in this direction are promising. First, in the advanced stage of periodontal disease, some miRNAs show dual regulation, which may be related to the fact that miRNAs can target and regulate multiple genes [32]. Second, miRNAs also play an important role in the differentiation of periodontal membrane stem cells into osteoblasts and have strong potential to become therapeutic targets for alveolar bone regeneration [33]. Third, miRNAs can regulate the immune response to infection, and drug development or drug combination from the perspective of immunity are new therapeutic strategies [15].

Therefore, our research can lay a certain foundation for exploring the mechanism of peri-implantitis to a certain extent, and is conducive to the screening of diagnostic markers and potential targets for treatment of peri-implantitis. It is still relatively novel to study the possible diagnostic methods of periodontitis and peri-implantitis through miRNA sequencing, which can help doctors make more accurate clinical diagnoses of diseases from more dimensions. Although the sample size of the study was small, further studies are needed to explore the functional role of specific miRNAs and their potential as therapeutic targets for diseases of periodontitis and peri-implantitis.

## Conclusions

In this study, we showed that GCF of PMSII displays a unique profile of miRNAs, and it may lay the foundation for future research on the molecular mechanism of PMSII. Our findings also suggest that miRNA detection in PMCF/CGF may serve as a novel diagnostic tool that circumvents the invasive procedure to obtain biopsy samples of gingival tissues to diagnose PMSII. Such as miR-4291, miR-1245b-3p and miR-1825 may be used as diagnostic markers and potential therapeutic targets for PMSII.

## Data Availability

The data that support the findings of this study are available from the corresponding author upon reasonable request. The raw data files of high-throughput sequencing were uploaded to https://zenodo.org/.DOI10.5281/zenodo.5733523.

## Abbreviations

miRNA: microRNA
PMICF: Peri-miniscrew implant crevicular fluid
NGS: Next-generation sequencing
GCF: Gingival crevicular fluid
qRT-PCR: Quantitative real-time PCR
GO: Gene Ontology
KEGG: Kyoto Encyclopedia of Genes and Genomes
WHO: World Health Organization
PMCF: Peri-implant crevicular fluid
FOXO: Forkhead box O
